# Strategic Design of Biocompatible, Glistening-Free, and Foldable Artificial Intraocular Lenses Based on Hydro-Amphiphilic Ternary Copolymers

**DOI:** 10.1021/acs.biomac.5c00588

**Published:** 2025-06-17

**Authors:** Cheng-Ti Hu, Zhi-Xuan Liang, Jhen-Yu Luo, Po-Hsun Chiu, Annabelle I. Day, Chih-Chen Hsieh, Po-Jen Shih, Jia-Han Li, Bo-I Kuo, I-Jong Wang, Jia-Yush Yen, Chi-An Dai

**Affiliations:** † Department of Chemical Engineering, 33561 National Taiwan University, Taipei 10617, Taiwan; ‡ Institute of Polymer Science and Engineering, 33561 National Taiwan University, Taipei 10617, Taiwan; § Department of Biomedical Engineering, 33561 National Taiwan University, Taipei 10617, Taiwan; ∥ Department of Engineering Science and Ocean Engineering, 33561 National Taiwan University, Taipei 10617, Taiwan; ⊥ Institute of Neuroscience, 34914 National Yang-Ming Chiao-Tung University, Taipei 112304, Taiwan; # Department of Ophthalmology, 38006 National Taiwan University Hospital, Taipei 100225, Taiwan; ∇ College of Medicine, 33561 National Taiwan University, Taipei 100233, Taiwan; ○ Department of Mechanical Engineering, 34878 National Taiwan University of Science and Technology, Taipei 106335, Taiwan

## Abstract

The increasing prevalence of cataracts underscores the urgent need for intraocular lens (IOL) materials that provide optical clarity, foldability, glistening resistance, and long-term biocompatibility. In this study, we developed hydro-amphiphilic ternary copolymers composed of styrene, hydroxyethyl methacrylate (HEMA), and poly­(ethylene glycol) phenyl ether acrylate (PEGPEA) to address these requirements. This rational design integrates the strength and refractive index of hydrophobic styrene with the flexibility and hydrophilicity of HEMA and PEGPEA. The optimized formulation (H3), comprising 50 wt % HEMA, 30 wt % PEGPEA, and 20 wt % styrene, showed excellent transparency after accelerated aging, sufficient modulus and elongation for safe surgical handling, and low cytotoxicity in CCK-8 assays. Cytokine analyses revealed no significant inflammatory response compared to a commercial hydrophobic acrylic IOL. These findings highlight hydro-amphiphilic copolymers as a promising next-generation material platform for IOLs, offering a biocompatible, glistening-free, and foldable solution for enhanced surgical outcomes and long-term patient satisfaction.

## Graphical abstract

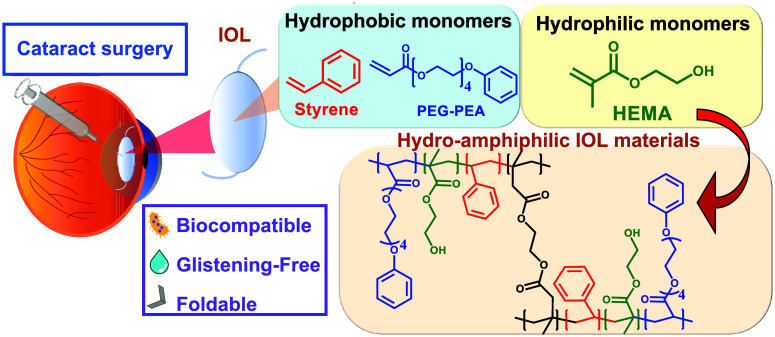

## 1 Introduction

Cataracts remain one of the primary causes of visual impairment and blindness across the globe. As a result, cataract surgery has become a commonly performed and standardized medical procedure.^1–3^ Contemporary surgical approaches—such as phacoemulsification and manual small-incision techniques—enable vision restoration by replacing the clouded natural lens with an artificial intraocular lens (IOL).^4,5^ The performance of IOLs relies on the harmonious optimization of their optical properties, mechanical characteristics, and biocompatibility.^6^ Over time, various materials have been used in IOL fabrication, from rigid glass and poly­(methyl methacrylate) (PMMA) to more flexible silicone-based materials.^7–9^ However, the rigidity of PMMA and glass lenses demands larger surgical incisions, thereby elevating the risk of postoperative astigmatism, whereas the low refractive indices of conventional silicone materials compel the use of thicker lenses.^10–13^ To overcome these limitations, acrylic copolymers have emerged as promising alternatives.

Acrylic IOLs are typically divided into hydrophobic and hydrophilic categories according to their material properties.^14–16^ Hydrophilic acrylics, derived from hydroxyl-containing monomers, exhibit high water content, excellent biocompatibility, and a reduced inflammatory response.^17–19^ However, their excessive water content decreases refractive indices and increases the risk of posterior capsule opacification (PCO).^20,21^ In contrast, hydrophobic acrylics feature higher refractive indices and support sharp-edged designs that effectively reduce PCO.^7,22–24^ Nevertheless, their low water content renders them susceptible to glistening—microvacuoles caused by long-term water condensation within the lens, which scatter light and significantly impair visual quality.^25–27^ Additionally, IOL designs must meet essential requirements such as biocompatibility and accommodation to minimally invasive surgical procedures. This includes the ability to be stored in a folded state in a syringe and to unfold rapidly and reliably during implantation to ensure successful positioning in the eye. Therefore, strategic material selection in IOL design is crucial, optimizing optical properties while maintaining biocompatibility and mechanical integrity to ensure both foldability and structural stability.

Common strategies for advancing IOL materials to meet the required standards involve optimizing the types and concentrations of mainchain monomers and cross-linkers, as well as incorporating postfabrication modifications.^28–30^ For example, Xiang et al. developed a PPPE hydrophobic acrylic IOL system, achieving optimal glistening prevention with minimal EHMA while maintaining foldability at 37 °C. Further polydopamine coating and gentamicin sulfate loading could potentially prevent complications while maintaining biocompatibility.^31^ Similarly, Liu et al. synthesized poly­(EGPEMA-*co*-EA) copolymers cross-linked with PEGDA to fabricate foldable IOL prototypes, identifying an optimal EGPEMA/EA ratio of 7:3, which provided low elastic modulus, high stretchability, and a low glass transition temperature, allowing for effective folding. Subsequent long-term in vitro and in vivo biocompatibility tests also demonstrated optimal outcomes.^32^ More recently, Kim et al. systematically investigated the effects of cross-linker type and concentration on the performance of IOLs fabricated from hydrophobic POEA. The results showed that increasing the hydrophilicity of the cross-linker effectively reduced glistening formation. Moreover, GDGDA, with its long flexible alkyl chain, lowered the *T*_g_ below the typical operating temperature of 20 °C, preserving foldability.^33^ However, most existing studies have focused predominantly on hydrophobic monomers with minimal incorporation of hydrophilic modifiers, restricting their investigations to narrow compositional ranges and overlooking the potential advantages of systematically integrating hydrophobic and hydrophilic monomers across a broader spectrum of ratios. Furthermore, comprehensive studies on more refined systems—such as ternary copolymers that systematically evaluate the interplay among multiple monomers—remain scarce.

In this study, we introduce a class of hydro-amphiphilic ternary copolymers—poly­(styrene-*co*-2-hydroxyethyl methacrylate-*co*-poly­(ethylene glycol) phenyl ether acrylate) (poly­(St-HEMA-PEGPEA))—designed to reduce glistening formation while maintaining optical properties, mechanical properties and biocompatibility. Styrene increases hydrophobicity and refractive index, HEMA enhances hydrophilicity and biocompatibility, and PEGPEA, with its flexible ethylene oxide chains and benzene rings, improves softness and refractive index.^34–36^ A systematic investigation of these ternary copolymers was conducted to optimize their composition for IOL applications. Our findings reveal that a balanced composition—50 wt % HEMA, 30 wt % PEGPEA, and 20 wt % styrene—achieves a sufficient Young’s modulus and high break strain, ensuring foldability for injectable implantation. Additionally, the copolymer demonstrates excellent optical transmittance and minimal glistening formation, even after accelerated aging tests. This improvement is attributed to the hydrophilic HEMA, which enhances equilibrium water content. In vitro assessments confirm that the IOL prototype exhibits biocompatibility comparable to commercial IOLs, supporting its suitability for clinical applications. This study establishes hydro-amphiphilic ternary copolymers as promising next-generation IOL materials, offering a biocompatible, glistening-free, and foldable alternative with superior performance characteristics.

## 2 Experimental Section

### 2.1 Materials

Poly­(ethylene glycol) phenyl ether acrylate (PEGPEA) comprising a phenyl ether moiety linked to a tetra­(ethylene glycol) chain with a terminal acrylate group, and the photoinitiator 2-hydroxy-2-methylpropiophenone (HMPP), were procured from Sigma-Aldrich. Additionally, 2-hydroxyethyl methacrylate (HEMA), styrene, and the cross-linker ethylene glycol dimethacrylate (EGDMA) were sourced from Acros Organics. All monomers were used as received, without further purification.

### 2.2 Preparation of IOL Films

The fabrication process of intraocular lens (IOL) films is depicted in Figure 1, while the sample compositions were derived from a ternary composition diagram shown in Scheme 1. The corresponding sample designations are provided in the associated table. The samples were categorized into three primary series: the S series, the H series, and the P series. In the S series, the styrene content progressively increased from S1 to S3, accompanied by a corresponding decrease in HEMA and PEGPEA contents. Similarly, in the H series and P series, the HEMA and PEGPEA contents, respectively, increased along their designated sequences. To avoid confusion, the intersection of the three series was labeled as sample O, ensuring a consistent naming convention. To prepare the samples, precise amounts of styrene, 2-hydroxyethyl methacrylate (HEMA), and poly­(ethylene glycol) phenyl ether acrylate (PEGPEA) were measured according to the specified compositions in the ternary phase diagram. The monomers were then mixed with 1 wt % of the photoinitiator 2-hydroxy-2-methylpropiophenone (HMPP) and 4 wt % of the cross-linker ethylene glycol dimethacrylate (EGDMA) to form a homogeneous solution. The resulting mixture was poured into a custom-designed sandwich mold for photopolymerization. The mold consisted of a polytetrafluoroethylene (PTFE) film (0.5 or 1 mm thick, matching the intended sample thickness) with a 2 × 2 cm central opening as the middle layer, polyethylene terephthalate (PET, 80 μm) films as the upper and lower support layers, and double-sided tape sealing the interface between the PTFE and PET layers to prevent monomer leakage. Photopolymerization was initiated by exposing the mold to ultraviolet (UV) light (365 nm, 80 W) for 30 min through the transparent PET layer, with the UV source positioned 7 cm above the mold. The mold was then inverted, and an additional 30 min UV exposure was applied to ensure complete polymerization throughout the sample. Following polymerization, the cured samples were thoroughly washed and stored in water to remove any residual unreacted monomers before further analysis.

**Scheme 1:**
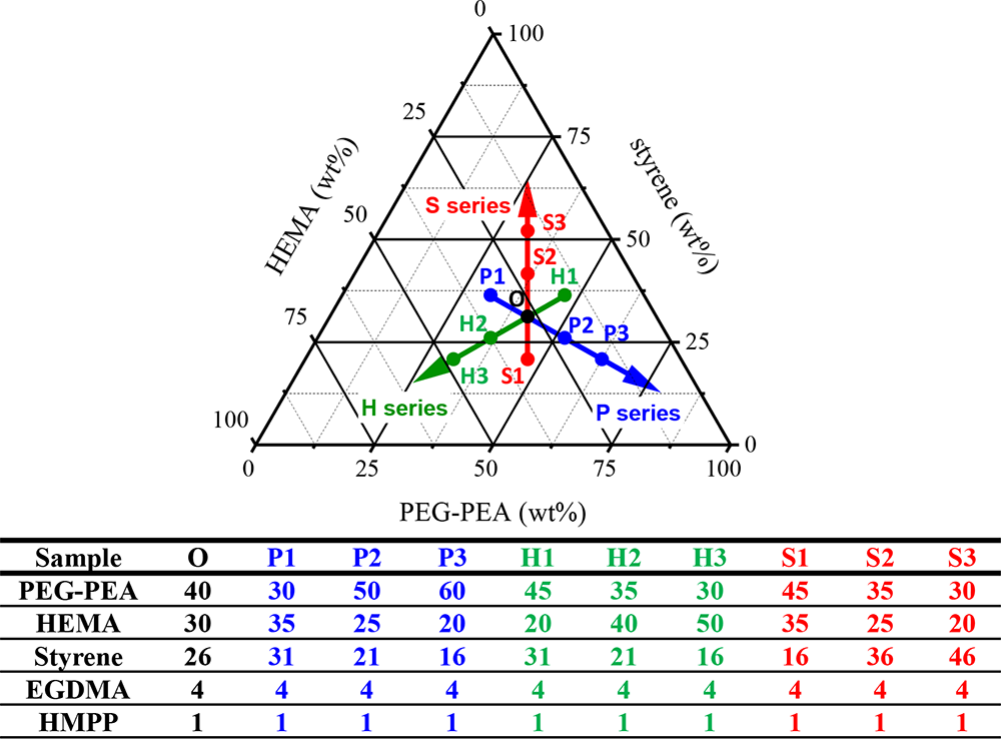
Ternary Composition Diagram of Styrene, HEMA, and PEGPEA, Serving as the Basis for the Preparation of IOL Samples^a^

a The sample designations are detailed in the accompanying table.

**Figure 1:**
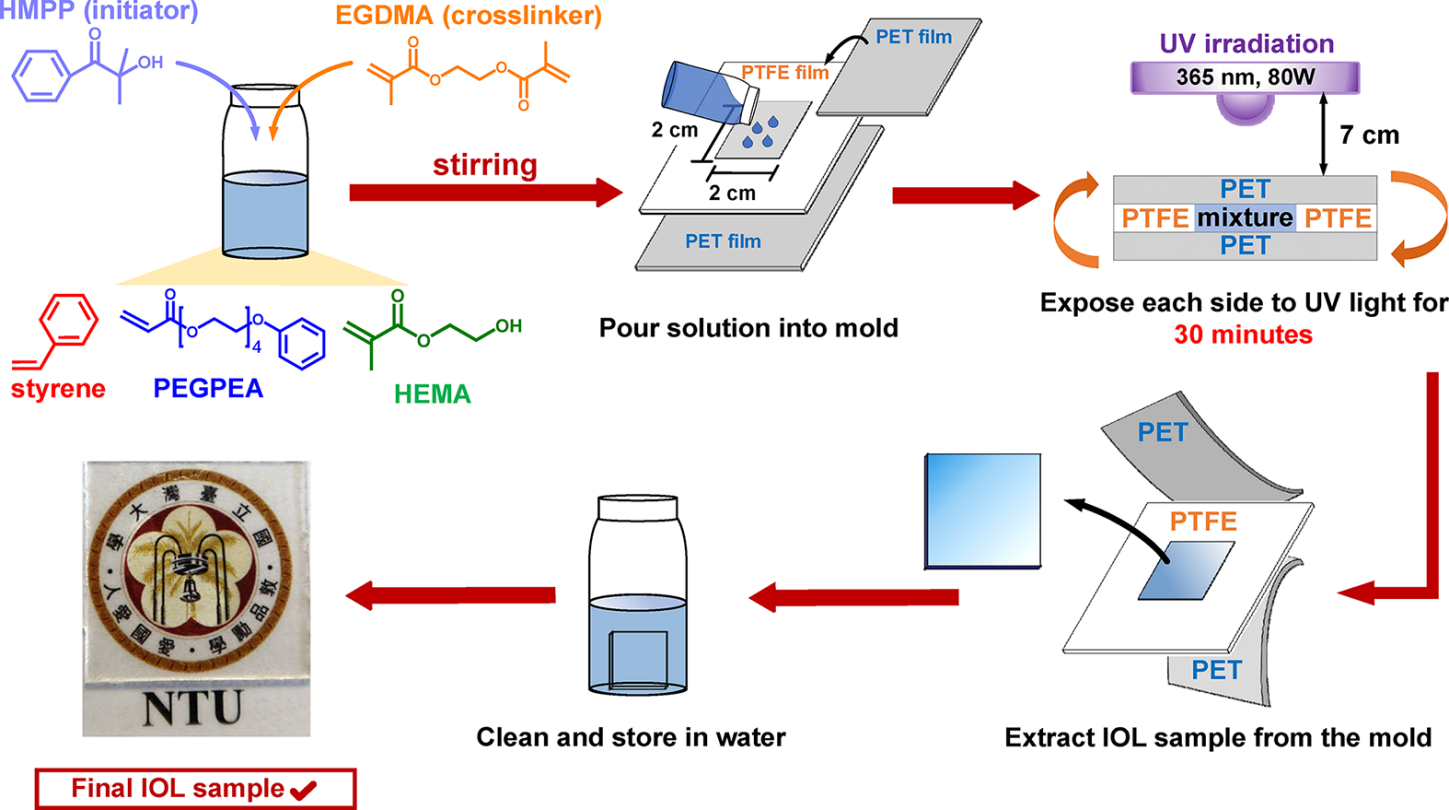
Schematic illustration of the fabrication process for multifunctional intraocular lens (IOL) films prepared from ternary monomer systems. Detailed preparation steps are described in the Section 2.

### 2.3 FTIR-ATR Measurements

To characterize the compositional differences among the IOL sample series, FTIR-ATR measurements were performed on selected dried and cured samples using a PerkinElmer Spectrum 100 Plus spectrometer equipped with a PIKE MIRacle single-reflection ATR accessory. Spectra were collected at a resolution of 1 cm^–1^ over the range of 650–4000 cm^–1^, and each spectrum was averaged over 16 scans.

### 2.4 Mechanical Property Measurements

Water-saturated IOL specimens (dimensions: 20 × 10 × 1 mm) were evaluated for their mechanical properties at room temperature using an MTS Criterion universal testing machine with a 250 N load cell. Tensile testing was performed at a constant elongation rate of 10 mm/min. The Young’s modulus was calculated from the linear portion of the stress–strain curve corresponding to ≤ 1% strain. Tensile strength and break strain were also recorded. All measurements were performed in triplicate for reproducibility.

### 2.5 Folding/Unfolding Measurements

The shape memory behavior and foldability of water-saturated IOL samples (10 × 10 × 0.5 mm) were evaluated using a folding test conducted at room temperature. Each sample was carefully folded in half using tweezers, reducing its size to 5 mm, while ensuring minimal pressure was applied to avoid excessive flattening. The folded sample was held in this state for 10 s before being released, at which point a timer was started. The timer was stopped when the sample unfolded from the folded length of 5 mm to 90% of its original length (i.e., 9 mm). The time required for this unfolding was recorded as the foldability.^37^ Results represent the average of three specimens.

### 2.6 Thermomechanical Properties

Dynamic mechanical analysis (DMA) of water-saturated IOL samples was performed using a HITACHI 7100 analyzer to determine the glass transition temperature (*T*_g_) and tensile storage modulus (*E*′). Tensile-mode testing was conducted on specimens (20 × 5 × 0.5 mm) using a 5 mm gauge length, with a temperature ramp rate of 3 °C/min and a test frequency of 1 Hz, across a temperature range spanning from – 20 to 120 °C.

### 2.7 Water Content (WC %)

IOL samples were immersed in water for 3 days to ensure full saturation. Wet weights (*W*_s_) of the samples were recorded after gently blotting surface water. Samples were subsequently dried at 50 °C under vacuum, and their weights were periodically monitored until a constant value was obtained. The final stabilized weight was designated as the dry weight (*W*_d_). Water content was calculated using the equation:

$$\mathrm{WC}\;\%=\frac{W_{s}-W_{d}}{W_{s}}\times 100\%$$

### 2.8 Optical Properties

The light transmittance spectra of water-saturated intraocular lenses (IOLs) were obtained using an Agilent CARY 300 UV–vis spectrophotometer over the 280–800 nm range, with particular emphasis on the visible spectrum (380–750 nm) for transmittance analysis. Refractive indices were measured using an ATAGO ABBE T3 refractometer. Sample thickness was maintained at 0.5 mm for all tests.

### 2.9 Glistening Formation Analysis

Glistening formation was evaluated in samples subjected to accelerated aging (1 day and 7 days in water at 50 °C) using dark field microscopy at 100× magnification. Central regions of the samples were analyzed for glistening evolution.

### 2.10 Cells and In Vitro IOL Cell Culture

Human lens epithelial cells (HLEB3; ATCC, Rockville, MD) were cultured in Dulbecco’s Modified Eagle’s Medium (DMEM; Gibco, USA) supplemented by 10% fetal bovine serum (FBS; Invitrogen, CA, USA) at 37 °C in a humidified incubator containing 95% air and 5% CO_2_. Upon reaching approximately 80% confluence, the cells were detached using 0.05% trypsin and 0.02% EDTA, and subcultured every 3 days. Prior to seeding, the cells were resuspended to a final concentration of $2\times 10^{5}$ cells/mL. Under sterile conditions, each intraocular lens (IOL) was positioned at the bottom of a 24-well plate, and 1 mL of the prepared cell suspension was gently added to fully cover the surface of the IOL.

### 2.11 Cell Adhesion and Proliferation

Cell adhesion and viability on IOL surfaces were assessed using the CCK8 assay (Vazyme, CN) per the manufacturer’s protocol. After a 24-h incubation, IOLs were transferred to new 96-well plates, followed by the addition of complete medium and CCK8 solution. Absorbance at 450 nm was measured using a microplate reader (Sunrise, Tecan), and results were expressed as the relative number of adherent cells. All experiments were conducted in triplicate.

### 2.12 Methods for RNA Isolation and Quantification of Target mRNA Expression

RNA was isolated utilizing the RNA Isolater Total RNA Extraction Reagent (Vazyme, CN). Subsequently, HiScript III RT SuperMix for qPCR (+gDNA wiper) (Vazyme, CN) was employed for cDNA synthesis, following the instructions provided by the manufacturer. The reaction was carried out by incubating the mixture at 37 °C for 15 min, followed by heating at 85 °C for 5 s to complete the reverse transcription. Exactly 1000 ng of total RNA was used per reaction. The generated cDNA was then subjected to quantitative PCR amplification using 20X SYBR Green gene expression assays targeting TGFB1, TNF, IL6, and ACTB (Topgen Biotech., TW). Primer sequences and assay details are provided in Table S1. Reactions were set up using ChamQ Universal SYBR qPCR Master Mix (Vazyme, CN) in a final volume of 10 μL, and amplification was conducted on the StepOne Plus Real-Time PCR System (Applied Biosystems, USA), strictly adhering to the protocol recommended by the manufacturer. Each sample, which contained 20 ng of cDNA, was subjected to triplicate analysis together with corresponding nontemplate controls (NTCs). Gene amplification results were normalized against ACTB expression, and relative expression levels—expressed in arbitrary units (a.u.)—were calculated using the 2­(-Delta Delta C­(T)) method. Ct values obtained from quantitative PCR showed a variability coefficient of less than 2% relative to the mean measurements.

### 2.13 Western Blot Analysis

After electrophoresis, protein samples were transferred onto PVDF membranes using a Hoefer Wet Tank Transfer system, operated at 210 mA for 1 h at room temperature. The membranes were then incubated with BlockPRO protein-free Blocking Buffer (BF01, Visual Protein, Taiwan) for 1 h at room temperature to minimize nonspecific binding. Following blocking, membranes were exposed to the corresponding primary antibodies and left to incubate overnight at 4 °C. Before and after treatment with the secondary antibody (1 hour at room temperature), the membranes were washed three times in TBST (0.05% Tween-20), with each wash step lasting 10 min.

## 3 Results and Discussion

### 3.1 Chemical and Mechanical Properties of the IOL Materials

The fabrication process of the IOL films is illustrated in Figure 1. A homogeneous mixture comprising HEMA, PEGPEA, styrene, a photoinitiator, and a cross-linker was cast into a custom-designed sandwich mold composed of PTFE and PET films, followed by photopolymerization under UV exposure from both sides. After polymerization, the films were thoroughly washed with deionized water to remove any unreacted monomers. To assess both chemical composition and material properties, the cured samples were divided into two groups: selected specimens were dried and used for FTIR-ATR analysis, while the remaining samples were stored in water to maintain equilibrium hydration prior to physical and mechanical property testing.

To confirm the successful incorporation of each monomer and to qualitatively reflect formulation-dependent compositional variation, FTIR-ATR spectra were collected from three representative dried samples—H3, P3, and S3—each formulated with the highest proportion of HEMA, PEGPEA, and styrene, respectively, for comparative analysis. The spectra, presented in Figure S1 of Supporting Information, exhibited three characteristic absorption regions: a broad O–H stretching band around 3400 cm^–1^ associated with HEMA; a C=O stretching band near 1720 cm^–1^ corresponding to ester functionalities from both HEMA and PEGPEA; and aromatic C=C stretching vibrations appearing at 1480 and 1450 cm^–1^, originating from the benzene rings of styrene and the phenyl ether group linked to a tetra­(ethylene glycol) chain in PEGPEA.^38–40^ The presence of these key spectral features across all three samples confirmed the successful incorporation of the targeted monomer units into the polymer networks. In addition, relative differences in peak intensities reflected formulation-dependent trends in monomer composition. The O–H signal was most prominent in H3 and diminished in P3 and S3, consistent with decreasing HEMA content. The C=O band appeared stronger in both H3 and P3, due to their higher proportions of ester-bearing monomers, and weaker in S3. Likewise, the C=C stretching bands were more clearly distinguishable in S3 and P3 than in H3, aligning with their greater aromatic content. These spectral trends collectively validated the compositional variation across the sample series and served as a qualitative foundation for subsequent correlation with material properties.

Building on the compositional analysis from FTIR-ATR, mechanical testing was subsequently carried out to investigate the structure–property relationship among the IOL samples. To initially assess their mechanical performance under physiologically relevant conditions, simulating the intraocular environment, the samples were tested in the water-saturated state without prior drying. Figure 2a–c presents the stress–strain curves of the prepared IOL specimens, highlighting the significant impact of monomer composition on mechanical behavior. The styrene-rich sample (S3) exhibits brittleness, characterized by high tensile strength but low break strain. In contrast, the PEGPEA-rich sample (P3) is extremely soft, displaying a low modulus, low break strain, and consequently, low overall tensile strength. The HEMA-rich sample (H3) demonstrates a high break strain of approximately 100% along with a moderate modulus, indicating balanced mechanical properties. To further evaluate the influence of each monomer on the mechanical behavior of the formulations and their potential application in intraocular lenses (IOLs), the Young’s modulus, break strain, and tensile strength of each composition are summarized in Figure 2d–f. In the P1 to P3 series, an increase in the concentration of poly­(ethylene glycol) phenyl ether acrylate (PEGPEA) resulted in significant reductions in Young’s modulus, break strain, and tensile strength. In contrast, the H1 to H3 series revealed that higher concentrations of hydroxyethyl methacrylate (HEMA) consistently led to a slight increase in Young’s modulus, while break strain decreased, with minimal variation in tensile strength. For S1 to S3 series, higher styrene concentrations, coupled with lower PEGPEA content, progressively enhanced modulus, tensile strength, and break strain from S1 to O to S2. Notably, sample S3, with the highest styrene content, displayed a marked increase in Young’s modulus and tensile strength, but with a dramatic decrease in break strain, consistent with its brittle nature.

**Figure 2:**
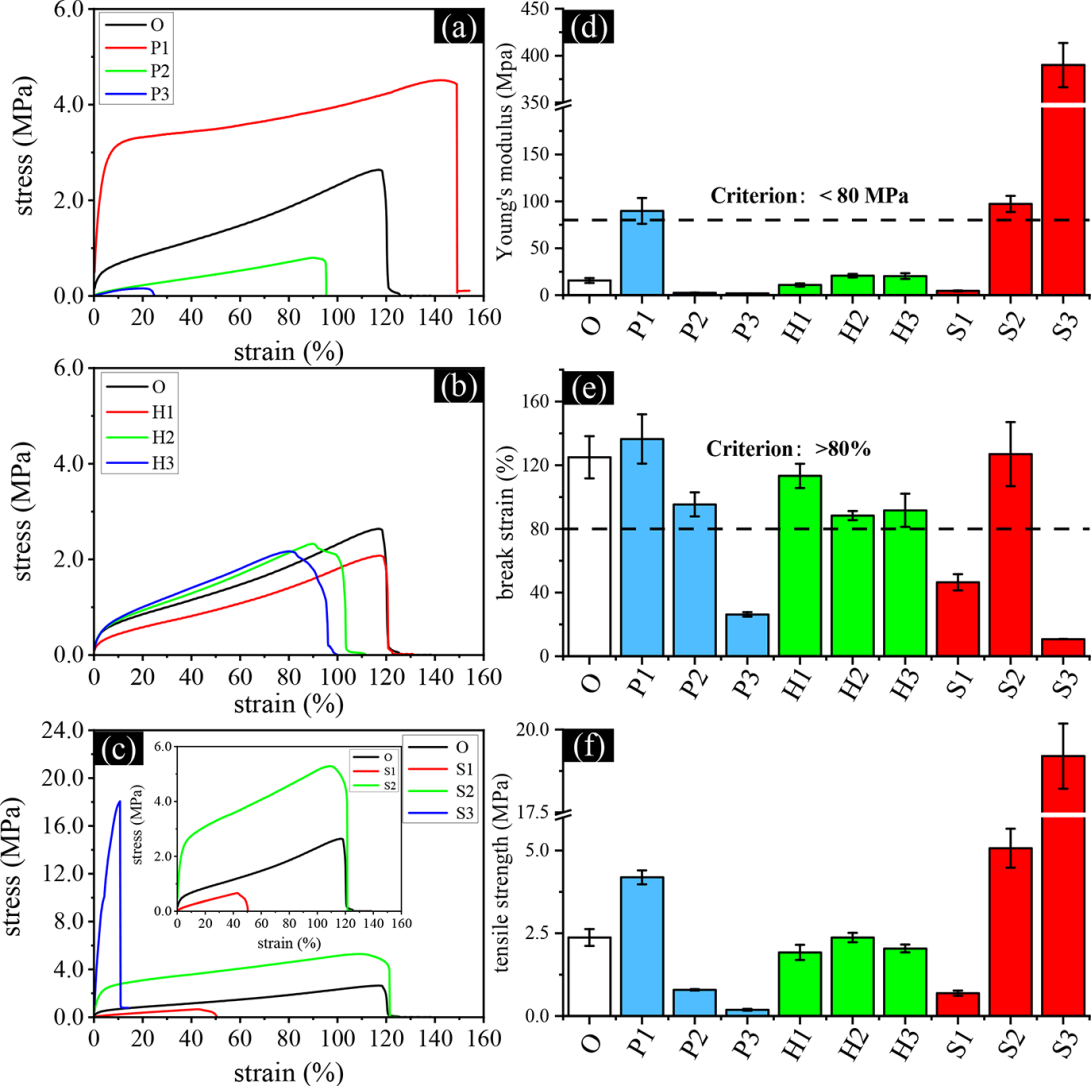
Mechanical properties of the prepared IOL films: (a–c) Stress–strain curves of the P, H, and S series, respectively, with summarized results for (d) Young’s modulus, (e) break strain, and (f) tensile strength. Due to the significantly larger tensile strength of S3, an inset in (c) highlights the stress–strain behavior of O, S1, and S2 on a comparable scale to other series.

The results presented above highlight key trends in mechanical properties influenced by each monomer, which are intrinsically governed by the molecular structure of the constituent monomers. PEGPEA consists of a flexible poly­(ethylene glycol) (PEG) segment and a rigid phenyl ether acrylate moiety, undergoing polymerization via acrylate cross-linking. As PEGPEA concentration increases, the incorporation of soft PEG segments rises, leading to a reduction in cross-link density and intermolecular interactions. This, in turn, increases free volume and decreases the polymer’s modulus. Furthermore, the reduction in cross-link density compromises network integrity and load transfer efficiency, resulting in premature failure under stress and a lower break strain. The combined effects of reduced modulus and break strain with increasing PEGPEA content ultimately lead to a significant decline in tensile strength. In contrast, styrene exhibits the opposite effect. The presence of benzyl side groups restricts molecular motion within polymer chains under external stress, progressively increasing Young’s modulus and tensile strength. In the S series, the break strain initially increased with rising styrene content (and decreasing PEGPEA content), attributed to the formation of a stronger yet flexible network structure from S1 to O to S2, which enhanced the material’s elastic behavior. However, at the highest styrene concentration (S3), the material became brittle, leading to a significant decrease in break strain. In the H series, an increase in HEMA content facilitated hydrogen bond formation via hydroxyl groups, thereby producing denser networks and slightly elevating Young’s modulus.

At low to moderate HEMA levels (from H1 to O), the moderately enhanced network compactness improved the break strain. However, at higher HEMA levels (from O to H3), the increased network rigidity slightly reduced the break strain, making the material marginally brittle. Nonetheless, the impact of HEMA on mechanical properties remained relatively modest compared to the other two components, as the increase in HEMA content was offset by reductions in PEGPEA and styrene, which balanced their respective contributions to the material’s overall behavior.

To ensure that IOL materials are both flexible and durable for cataract surgery and long-term use, specific mechanical property criteria were established. In this study, we followed the practical commercial standards outlined in the ISO regulations and adopted by many IOL manufacturers.^41^ Samples with a Young’s modulus not exceeding 80 MPa and a break strain exceeding 80% were considered potential candidate materials for further testing across three distinct series. As a result, samples O, P2, H1, H2, and H3 were determined to meet these standards. Notably, none of the samples from the S series were selected. Sample S1, with a high concentration of PEGPEA, demonstrated reduced cross-link density, impairing network integrity and resulting in low break strain. As styrene content increased from S2 to S3, both samples exhibited excessively high Young’s modulus, failing to meet the standard. In the P series, only sample P2 met the requirements, as sample P3 exhibited significantly lower break strain due to high PEGPEA content, which compromised network integrity, and sample P1, with high styrene content, exhibited a higher Young’s modulus. In contrast, the H series achieved a more balanced material composition, with a gradual increase in HEMA content and a reduction in both styrene and PEGPEA, resulting in a material that effectively balanced flexibility and strength, thus meeting the mechanical requirements for IOL applications. This underscores the importance of precise compositional control in optimizing material performance.

### 3.2 Dynamic Mechanical Property of the IOL Materials

During the implantation process, intraocular lenses (IOLs) undergo rapid dynamic transformations in both shape and temperature—transitioning from a folded, taco-like configuration within the injector to a disk-like form in the anterior chamber, while shifting from room temperature to body temperature. It is therefore critical to evaluate their thermomechanical properties, which govern their ability to withstand such deformation during thermal progression, ensuring effective folding and unfolding throughout the implantation process. To examine critical parameters, including the glass transition temperature (*T*_g_) and the modulus as a function of temperature, dynamic mechanical analysis was conducted to assess the viscoelastic behavior of water-saturated IOL samples. Figure 3a–c presents the tanδ-temperature curves for the analyzed samples. The results demonstrate that variations in composition within each series lead to distinct primary peak temperatures corresponding to α-relaxations, which are indicative of large-scale cooperative motions of polymer backbone and serve as the principal markers of *T*_g_. Furthermore, certain samples, particularly those in the H-series, exhibit shoulder features or secondary peaks at lower temperatures, attributed to β-relaxations. These β-relaxations are associated with the movement of side groups or small segments of the main polymer chain rather than the entire backbone.^42,43^ This observed behavior underscores the influence of different monomer compositions in shaping the material’s mechanical properties.

**Figure 3:**
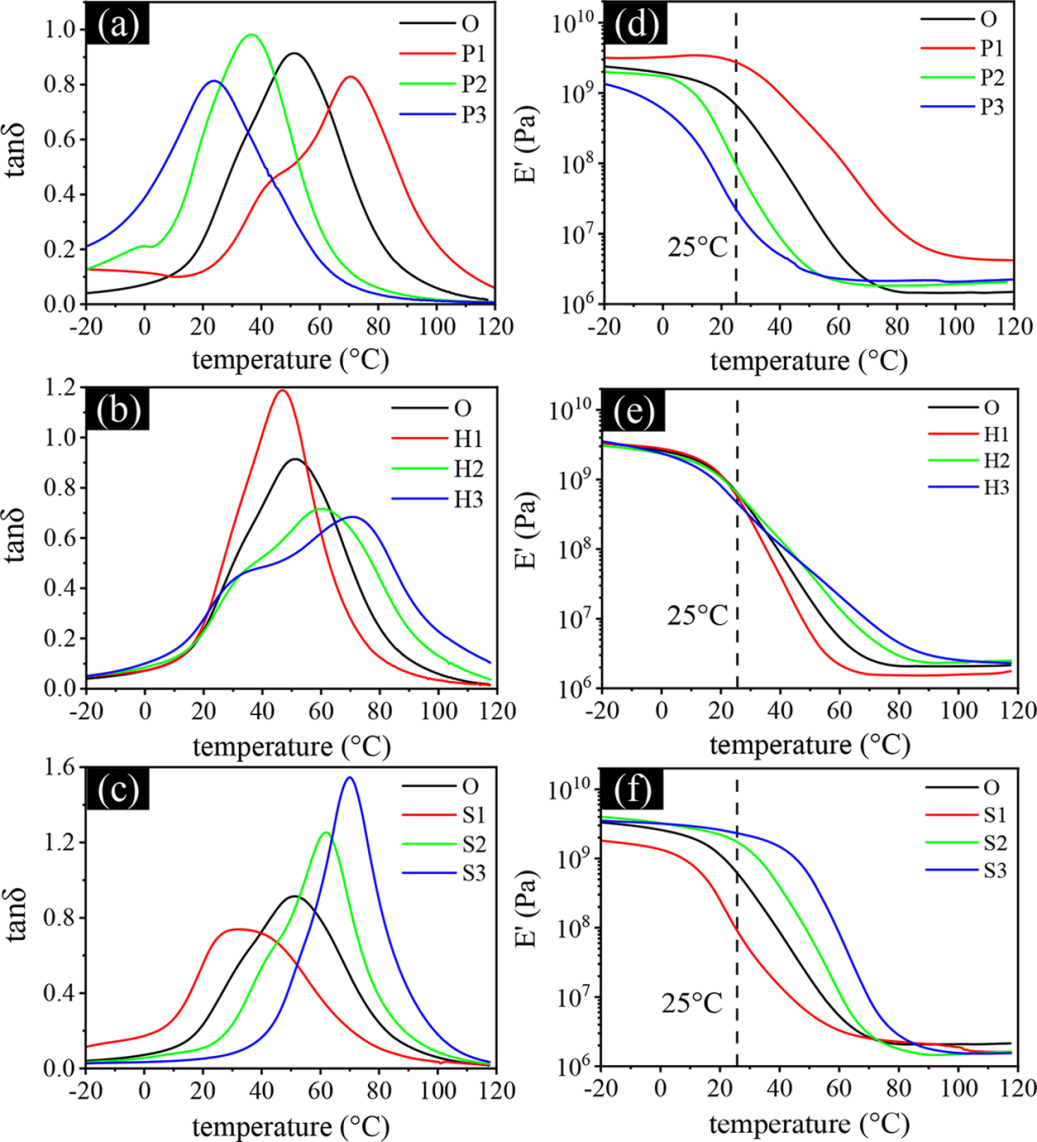
(a–c) Tan δ and (d–f) storage modulus versus temperature curves of water-saturated IOL films.

In the P series (Figure 3a), increasing the PEGPEA concentration from P1 to P3 consistently reduced the *T*_g_ of the samples. This reduction was attributed to the flexible ethylene oxide side chains in PEGPEA, which increased surrounding free volume and reduced the content of rigid styrene. Conversely, in the H series (Figure 3b), *T*_g_ gradually increased with higher HEMA incorporation due to the formation of a stronger hydrogen-bonding network involving HEMA’s hydroxyl groups, which restricted molecular motion. Similarly, the S series (Figure 3c) showed a rise in *T*_g_ with increased styrene content, as the rigid benzyl side groups further restricted molecular mobility. Moreover, enhanced β-relaxations in HEMA-rich samples were likely linked to increased water absorption, as water molecules acted as plasticizers. This effect improved the mobility of local polymer chains within the copolymers, intensifying β-relaxation signals—a characteristic behavior of hygroscopic materials upon water absorption.^44–47^ Such mobility could benefit foldability, as the mobile regions tend to recover their original shape during folding, while stiffer regions resist external stress without fracturing.^48,49^

In addition to tanδ curves, Figure 3d–f provides complementary storage modulus data as a function of temperature for the three series. All samples showed a significant decline in modulus at the onset of the glass transition, following a sequence within each series that aligns with the *T*_g_ trends observed in the tan δ results. For IOLs to perform effectively, they must balance sufficient flexibility with a moderately low modulus at operational temperatures near room temperature,^50,51^ enabling folding and unfolding without fracturing or sustaining irreversible damage during implantation. To facilitate data interpretation, auxiliary lines at 25 °C are marked in each figure.

The results suggest that most samples were in their glass transition region near room temperature, where increased chain mobility leads to a reduction in modulus and greater frictional energy dissipation. A moderate decrease in modulus and enhanced chain flexibility are expected to improve folding and unfolding performance while reducing the risk of mechanical failure. This highlights the necessity of aligning the material’s *T*_g_ with the operational temperature to achieve optimal performance. However, materials with extreme compositions posed significant challenges. Sample S3, which had the highest styrene content, remained in a glassy state at room temperature, resulting in a high elastic modulus (∼3 GPa) that rendered it unsuitable for folding or rapid unfolding. In contrast, sample P3, with the highest PEGPEA content, exhibited the lowest modulus (∼0.02 GPa), as it was near the terminal phase of the transition. While this characteristic may facilitate folding, it also made the material susceptible to flow-induced fracturing under external forces, thereby disqualifying it as a viable IOL candidate. These findings underscore the importance of achieving a precise balance in thermomechanical properties to ensure optimal IOL performance. The following section will validate these results through foldability tests, further examining the relationship between material composition, thermomechanical behavior, and functional performance under physiological conditions.

### 3.3 Foldability Assessment of the IOL Materials

To ensure reliable implantation, IOLs must remain stable in their folded state within the injector and unfold accurately and efficiently upon ejection. To assess this functionality and mitigate the risk of implant failure, the tensile and dynamic mechanical properties of the IOL samples were analyzed in the earlier sections. Subsequently, direct folding tests were performed to correlate these theoretical findings with practical outcomes. The post-test appearances of the samples are presented in Figure 4a–g, while Figure 4h summarizes the unfolding times required for the samples to recover 90% of their original length from a half-folded state. Certain samples are absent from the images (Figure 4a–g) due to their inability to restore their original shape after folding. This includes samples with excessively long unfolding times (>60 s, such as P1 and S3) and those that experienced permanent film rupture, such as P3. Moreover, although samples like H1 and S2 achieved the desired degree of unfolding, visible crease remained. Combined with the mechanical and viscoelastic properties discussed earlier, these findings highlight the critical role of polymer composition in determining folding capability.^52^ For the S series, increasing styrene concentration (from S1 to S3) raised the *T*_g_ of the samples, restricting polymer chain motion and resulting in a stiffer material, which prolonged unfolding times. Notably, S3 took over 60 s to reach the predefined recovery state, consistent with its glassy nature, significantly restricting chain mobility during unfolding recovery. This behavior aligns with its exceptionally high glass transition temperature (*T*_g_), which exceeds room temperature—the condition under which the folding experiment was conducted (Figure 3f)—as well as its high glassy-state storage modulus (Figure 3f). Meanwhile, S2, despite having a slightly lower *T*_g_ than S3 that enabled it to unfold more quickly, remained nearly in the glassy state when folded and developed visible creases or cracks. This was attributed to significant stress localization around the folded area, potentially exceeding its tensile strength and leading to local fracture. These observations highlight that effective folding and unfolding recovery require IOL materials with not only a sufficiently low *T*_g_ but also an appropriate modulus or strength to prevent cracking or rupture during deformation.

**Figure 4:**
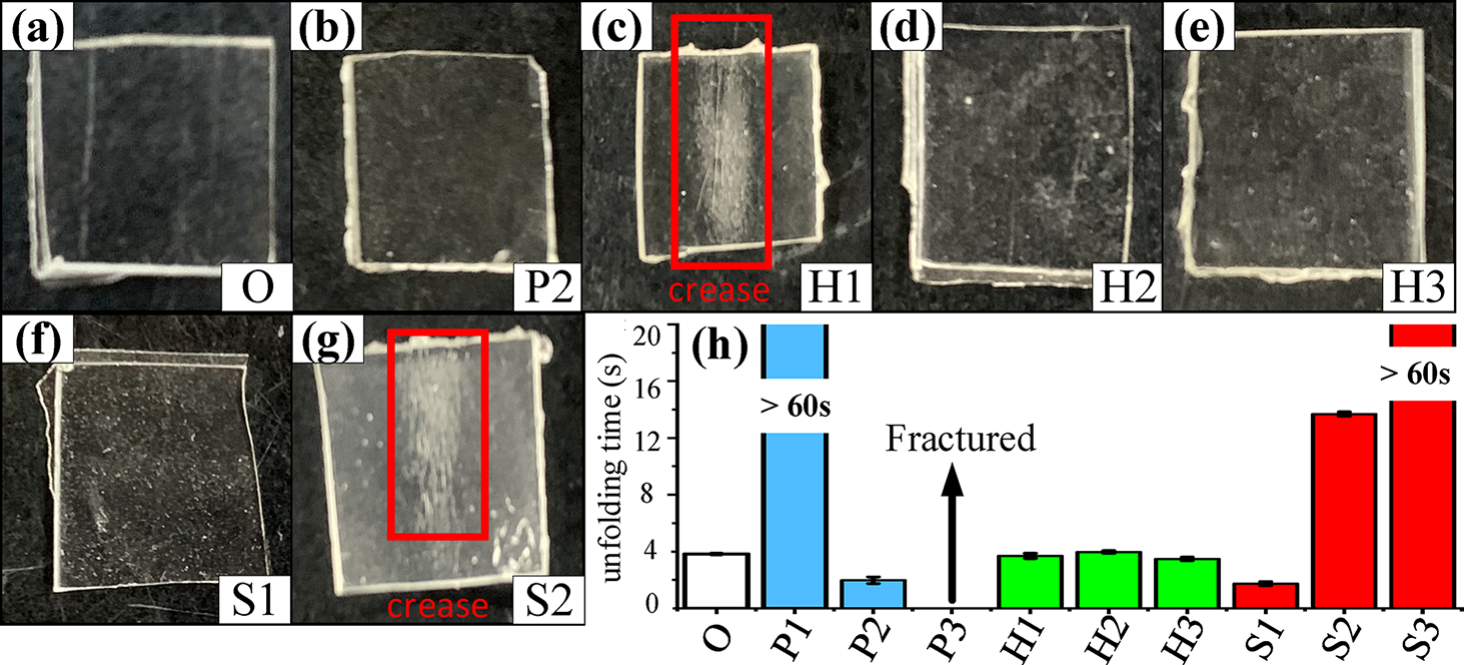
Foldability test results of fabricated IOL materials. (a–g) show the appearances of selected samples after folding tests, with unfolding times under 60 s and no permanent rupture. (h) Summary of unfolding time data for each tested sample.

In the P series, increasing PEGPEA content progressively lowered both *T*_g_ and modulus, resulting in shorter unfolding times, with P1 (>60 s) decreasing to approximately 2 s for P2. P1, which exhibited the highest *T*_g_ well above room temperature, remained in a glassy state, leading to extremely slow recovery, similar to S3. In contrast, the PEGPEA-rich sample, P3, fractured under external forces. This failure can be attributed to the high PEGPEA concentration, which reduced cross-link density, compromising network integrity and rendering it unable to withstand external stresses during folding. The rupture was further supported by its low tensile strength and break strain (Figure 2a). These findings underscore the importance of maintaining a moderately low modulus: while a lower modulus enhances flexibility, excessively low values compromise durability and structural integrity.

For the H series, the samples exhibited comparable mechanical properties, particularly in Young’s modulus (Figure 2d) and storage modulus at 25 °C (Figure 3e), resulting in similar unfolding times across the series. Their closely aligned glass transition temperatures ensured a consistent time scale for molecular chains to return to stable states, contributing to uniform unfolding behavior. Additionally, the previously observed β-relaxation, facilitated by water absorption, partially eased restrictions on polymer chain mobility, allowing faster recovery. This resulted in the H-series samples achieving the shortest average unfolding times (∼4 s), surpassing other series. However, such facilitation was less significant in sample H1 compared to the others, and its simultaneously lower tensile strength ultimately resulted in visible creases after folding, highlighting the critical role of tensile strength in resisting localized deformation and maintaining structural integrity.

According to the foldability tests, samples O, S1, P2, H2, and H3 were identified as suitable candidates for practical IOL applications. However, when taking the intersection with the mechanical property criteria, none of the samples in the S series satisfied both requirements. Samples S2 and S3, with their high styrene content, exhibited elevated moduli in both tensile and DMA tests, rendering them unsuitable. Conversely, sample S1, which incorporated a high concentration of PEGPEA, achieved the softness needed to pass the foldability test but suffered from reduced cross-link density and low break strain, raising concerns about long-term durability. A similar trend was observed in the P series. Extreme compositions, such as P1 (lowest PEGPEA content) and P3 (highest PEGPEA content), failed to meet the requirements, leaving only sample P2 as a viable candidate. Sample P3 showed low break strain and insufficient moduli, making it prone to fracture during folding, while P1 exhibited excessively high moduli that failed the mechanical standard and caused creasing upon folding. In contrast, the H-series samples demonstrated a more balanced performance across the three series. Increasing HEMA content from H1 to H3, accompanied by a reduction in styrene and PEGPEA, produced materials that effectively balanced moduli and strength, enhancing both mechanical and folding performances. However, sample H1, with its lower HEMA content, lacked sufficient strength to endure complete folding and unfolding without creasing. This highlights the importance of precise compositional control in optimizing material performance.

### 3.4 Optical Performances of the IOL Materials

To evaluate the optical transmittance, which reflects the achievable optical clarity of the intraocular lens (IOL) materials, the ultraviolet–visible (UV–vis) light transmittance spectra of the IOL samples are shown in Figure 5. Additionally, optical images illustrating their clarity are provided in Figure S2 of the Supporting Information. UV–vis analysis revealed that all samples exhibited excellent light transmittance, exceeding 92% across the majority of the visible light spectrum (380–750 nm), with only minor variations among them. These slight differences in transmittance are likely attributable not to inherent compositional differences but rather to surface roughness induced during the demolding process from the film fabrication mold. This finding underscores the effectiveness of integrating hydrophilic and hydrophobic components in the optical materials, promoting homogeneity and reducing light scattering or opacity—factors commonly associated with diminished transmittance. The accompanying optical images of the IOL materials further corroborate these results, showing clear visualization of sharp details and accurate colors of the figures beneath the samples. Collectively, these findings highlight the superior optical clarity of the materials, reinforcing their potential suitability for IOL applications.

**Figure 5:**
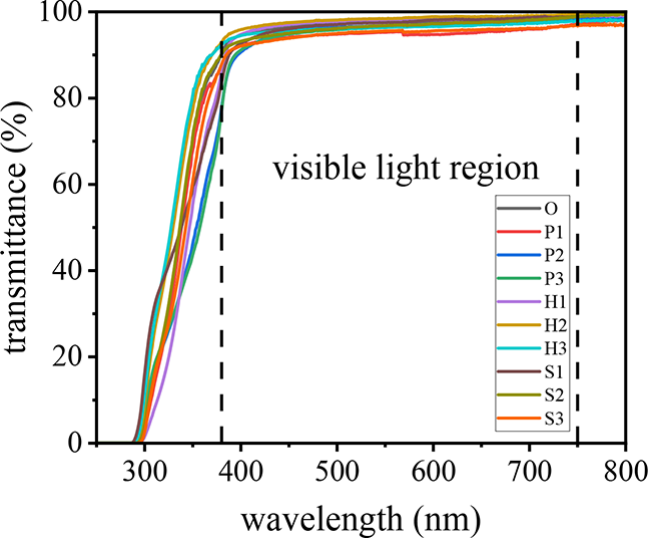
UV–vis optical transmittance spectra of the IOL samples.

### 3.5 Evaluation of Glistening Properties: Water Content and Optical Microscopy Measurements

The presence of glistening in intraocular lenses (IOLs) is linked to the development of microvacuoles, small fluid-filled inclusions within the lens matrix that induce light scattering and ultimately reduce visual clarity. This phenomenon is primarily attributed to fluid infiltration and phase separation, often exacerbated by temperature fluctuations during or after surgery, which influence water solubility within the material. To investigate the glistening-prevention capabilities of IOL materials, the water content of the fabricated samples was systematically analyzed. As illustrated in Figure 6a, the measured water content of the samples ranged from 3 to 8%. These levels are higher than those of typical hydrophobic IOL materials (0.1–0.5%) but lower than those of hydrophilic IOL materials (18–34%), aligning well with reported values for certain newly developed hybrid lenses (∼5%).^22^ Furthermore, the contribution of individual monomers to the total water content was evaluated across different sample series. In the H series, an increase in hydroxyethyl methacrylate (HEMA) concentration corresponded to higher water retention, attributed to the hydrophilic hydroxyl groups in HEMA, which enhance material hydrophilicity. Conversely, in the S series, the incorporation of hydrophobic styrene resulted in reduced water content as styrene concentration increased. In the P series, samples containing poly­(ethylene glycol) phenyl ether acrylate (PEGPEA) exhibited minimal variations in equilibrium water content, even with adjustments in PEGPEA levels. Notably, an increase in PEGPEA concentration led to a proportional decrease in styrene and HEMA composition, resulting in stable water content across the P series. The limited changes observed in the P series were attributed to the molecular characteristics of PEGPEA, which combines hydrophilic ethylene oxide chains with hydrophobic benzyl rings, as well as the reduced contributions of styrene and HEMA. These findings highlight the complex interplay of monomer composition and molecular design in influencing the water retention properties of IOL materials and their potential role in mitigating glistening.

**Figure 6:**
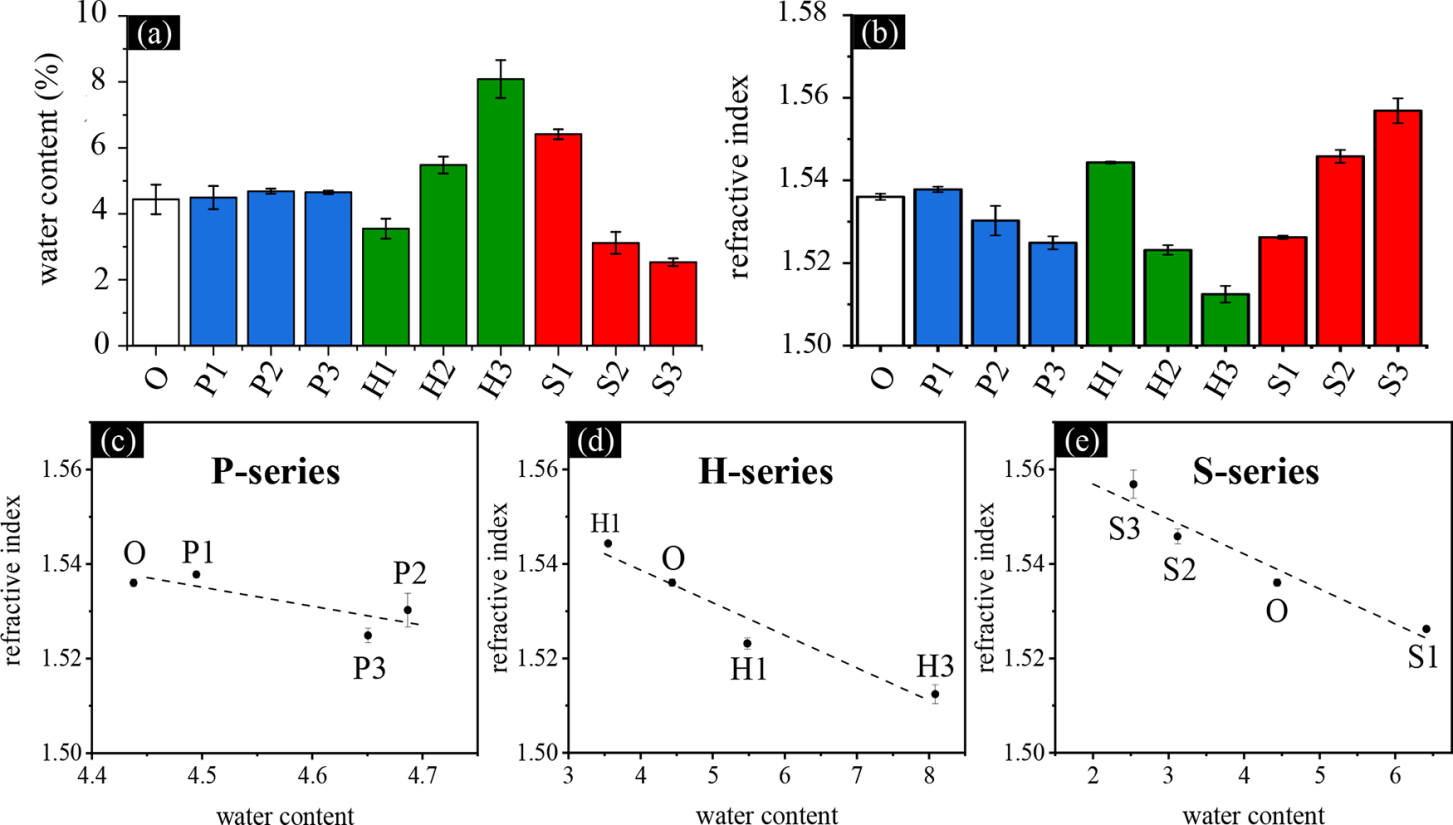
(a) Equilibrium water content and (b) refractive indices (RI) of the IOL samples. The relationship between RI and water content is summarized for (c) P-series, (d) H-series, and (e) S-series samples.

The refractive index (RI) values of the fabricated intraocular lens (IOL) samples are schematically presented in Figure 6b, showcasing RIs within the typical range for commercially available IOLs, between 1.45 and 1.55.^10,53,54^ From a material design perspective, the contributions of the constitutive components to the resulting RI can be categorized into two aspects: the inherent RI of individual components and the accompanying effects introduced by specific component interactions, e.g., water content. Regarding the former, HEMA and styrene demonstrated contrasting influences on RI: styrene (RI = 1.56) increased the RI of the materials, while HEMA (RI = 1.45) reduced it. Additionally, increasing PEGPEA concentration (RI = 1.50) led to a moderate reduction in RI. For the latter, equilibrium water content and the trade-off between components significantly influenced RI performance. Figure 6c–e illustrate the inverse relationship between RI and water content across all series. These combined effects highlight how precise formulation adjustments can effectively manipulate RI. In the S-series samples, a higher styrene content corresponded to a gradual increase in RI from S1 to S3. This increase was primarily due to the inherently high RI of the styrene monomer and its hydrophobic nature, which also reduced the water content of the material. Since water has a relatively low RI of approximately 1.3, this reduction in water content contributed significantly to the overall RI increase. In contrast, HEMA, with its lower RI and higher water retention capacity, tended to decrease the overall RI of the material. For PEGPEA, variations in water content with changes in its concentration were minimal, and its RI remained intermediate at 1.50. Consequently, incorporating PEGPEA produced only moderate changes in RI (Figure 6c), especially when compared to the more pronounced effects of HEMA and styrene (Figure 6d,e). Previous clinical studies have reported potential complications associated with high RIs, such as cat’s eye reflex, dysphotopsia, and glare.^5,55,56^ While an increased RI offers the advantage of achieving thinner lens designs with sufficient dioptric performance, the RI of IOL materials must be carefully optimized. In this study, samples O, P2, H2, and H3 were identified as optimal candidates for IOL materials, as they met the requirements for mechanical properties and foldability. These samples demonstrated moderately high RIs—greater than 1.5 to facilitate thinner structures—while remaining below the upper limit of commercially available IOLs (1.55).

In addition to the mechanical and optical properties previously discussed, glistening formation represents another critical factor affecting the long-term performance of intraocular lenses (IOLs). To evaluate this phenomenon, the IOL samples were subjected to accelerated aging tests at 50 °C for 1 and 7 days, following protocols commonly used to induce glistening formation by exposing materials to temperatures between 35 and 50 °C for several hours to days.^29,57^ These conditions are intended to simulate long-term intraocular environmental effects within a condensed time frame. The resulting microstructures were examined using dark-field optical microscopy.

Figure 7 presents micrographs of the samples following accelerated aging at 50 °C for 1 day. Among them, H1 and S3 exhibited prominent microvacuole-like features, highlighted in red frames, ranging from several to over a dozen micrometers in size. These structures appeared as bright, discoid spots with distinct optical boundaries and strong contrast against the surrounding matrix. In contrast, other samples only showed small, faint, and ill-defined bright spots scattered across the surface, which lacked uniformity in shape or contrast.

**Figure 7:**
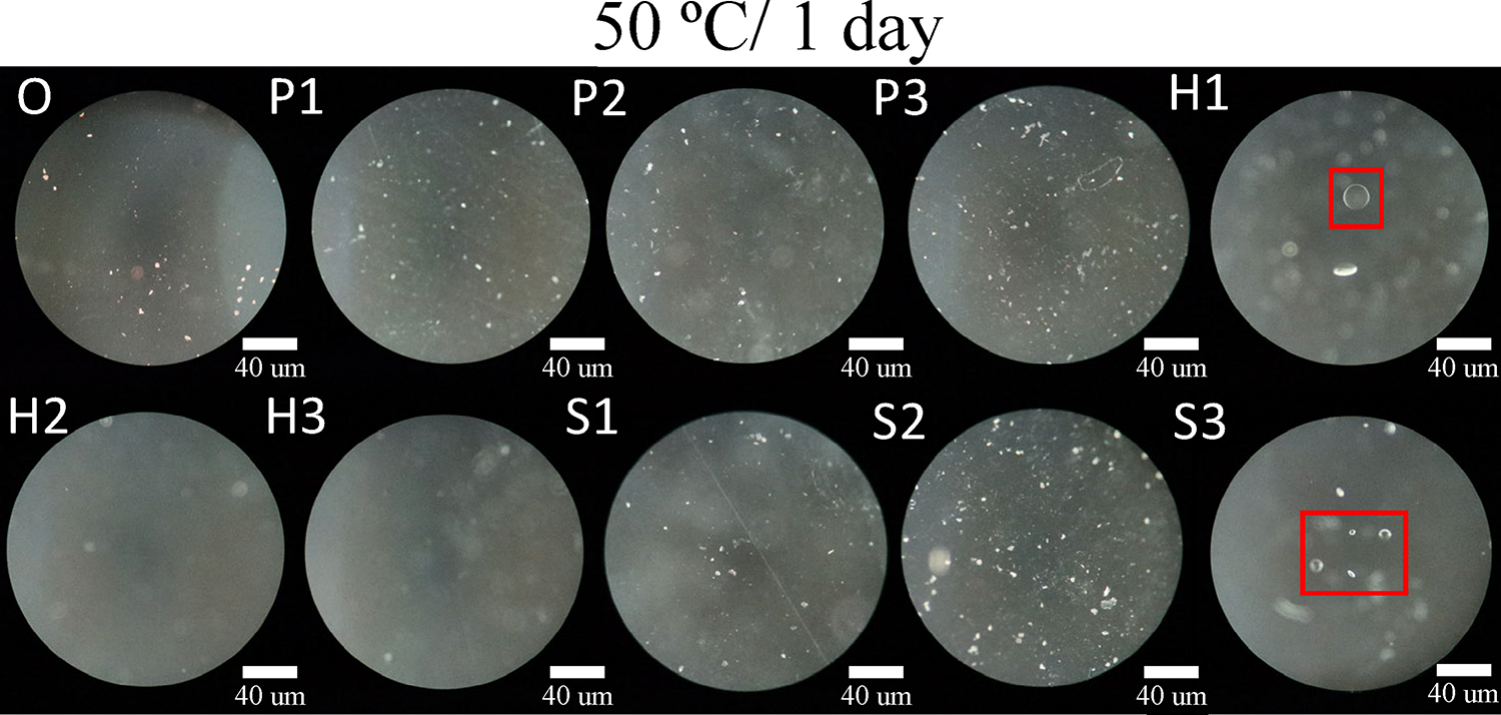
Dark field micrographs of IOL materials following accelerated aging at 50 °C for 1 day. The red frames highlight the formation of glistenings within the samples.

To clarify the criteria used to identify glistenings, we evaluated the observed features based on two primary parameters: size and morphology. According to previous findings, microvacuoles in the 6–25 μm range are most strongly associated with light scattering and visual degradation, and were thus classified as glistenings in this study.^58^ Furthermore, only features that exhibited a bright discoid appearance with distinct edges were included, as such morphology indicates a strong refractive index contrast between the polymer matrix and enclosed water. In contrast, small, vague, and poorly defined spots—as well as smaller bright specks or grain-like features—were excluded, as they were more likely attributable to surface imperfections or demolding artifacts. Based on these criteria, the vacuoles observed in H1 and S3 were classified as glistenings, while the features in other samples were not. These two samples, which exhibited the lowest equilibrium water content in prior tests, demonstrated a strong correlation between water uptake capacity and the suppression of glistening formation. The hydrophilicity of the materials was primarily attributed to the hydroxyl groups in HEMA and the polar ethylene oxide side chains in PEGPEA. During aging at temperatures near or above the glass transition temperature (*T*_g_) of the materials, where the bulk polymer matrix undergoes large-scale molecular motion, water molecules interacted differently depending on the material’s hydrophilic–hydrophobic balance. Hydrophilic segments absorbed and distributed water more uniformly, while hydrophobic regions promoted local water exclusion and aggregation over time. Consequently, samples incorporating more hydrophilic monomers exhibited more homogeneous water dispersion and effectively suppressed microvacuole growth. This behavior was consistent with the significant glistening observed in H1 and S3, which had low HEMA and high styrene content, supporting the notion that insufficient hydrophilicity promotes glistening formation.

To further assess glistening resistance over time, the samples were subjected to extended accelerated aging for 7 days, as shown in Figure 8. In addition to the enhanced vacuole aggregation observed in H1 and S3, sample S2 also began to exhibit vacuole features that met the defined size and morphology criteria. The progression of glistening formation in these samples further confirmed that lower water content and insufficient hydrophilic monomer incorporation increase the likelihood of water microvacuole aggregation, especially under thermally accelerated aging conditions. These results reinforce the importance of balanced hydrophilic design in achieving glistening-free IOL materials.

**Figure 8:**
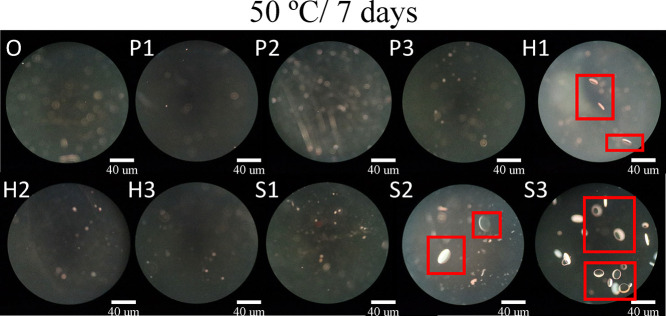
Dark field micrographs of IOL materials after accelerated aging at 50 °C for 7 days. The red frames highlight the formation of glistenings within the samples.

### 3.6 Assessment of the Biocompatibility of the IOL Materials

To evaluate the biocompatibility of the self-prepared intraocular lens (IOL) samples as potential candidates for IOL materials, we compared them to a commercially available IOL. The study included four prototype IOL materials (Samples O, P2, H2, and H3) and one commercial IOL (enVista MX60, Bausch & Lomb) as the control group. These prototype IOLs were cocultured with HLEB3 cells, and cell viability was subsequently determined utilizing the CCK-8 assay, as shown in Figure 9a. The control group exhibited the highest absorbance at 450 nm, followed by H3 and H2. No statistically significant differences in absorbance were observed among these three groups, indicating that H3 and H2 exhibited similar or lower cytotoxicity than the control. Cell counts on the IOL surfaces (Figure 9b) revealed the highest cell proliferation in the control group, with H3 showing the next highest proliferation. Evaluation of cell morphology on the surfaces of the control and H3 IOLs further confirmed appropriate HLEB3 cell growth on both materials (Figure 9c for the control group and Figure 9d for the sample H3). Based on these findings, H3 was selected for further cytokine evaluation.

**Figure 9:**
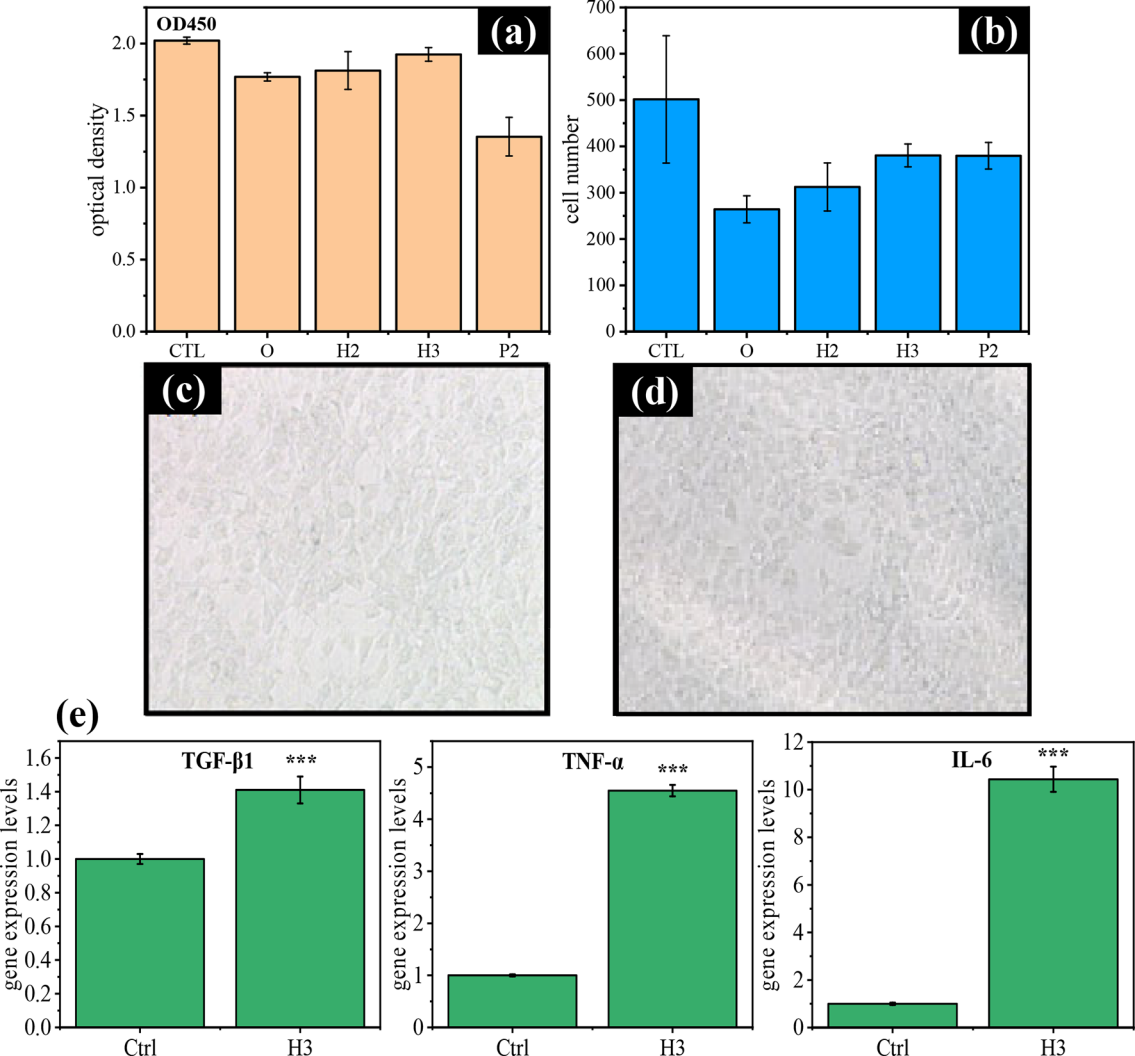
(a) CCK-8 assay results comparing IOL prototypes with the control group. (b) Quantification of cell density on IOL surfaces. (c,d) Cell morphology observed on the surfaces of the control group (c) and sample H3 (d). (e) Gene expression levels of TGF-β, TNF-α, and IL-6 in the control and H3 groups.

The mRNA expression levels of TGF-β, TNF-α, and IL-6 in HLEB3 cells were compared between the H3 and control groups, as shown in Figure 9e. The H3 group exhibited elevated mRNA levels for all three cytokines, suggesting a potential inflammatory response. However, as shown in Figure 10a–c, Western blot results and quantitative analyses of TGF-β, TNF-α, and IL-6 protein levels revealed no statistically significant differences between the H3 group and the commercial control. While the H3 group demonstrated increased cytokine mRNA expression, protein expression levels remained comparable to the control group. Taken together, these results highlight H3′s favorable combination of hydrophilic and hydrophobic properties, which provide optimal mechanical strength, optical clarity, and long-term usability. Moreover, H3 exhibited comparable in vitro biocompatibility to the commercially available IOLs. Therefore, H3 emerges as a promising candidate for future intraocular applications, offering an optimized pathway for advancing the artificial lens industry.

**Figure 10:**
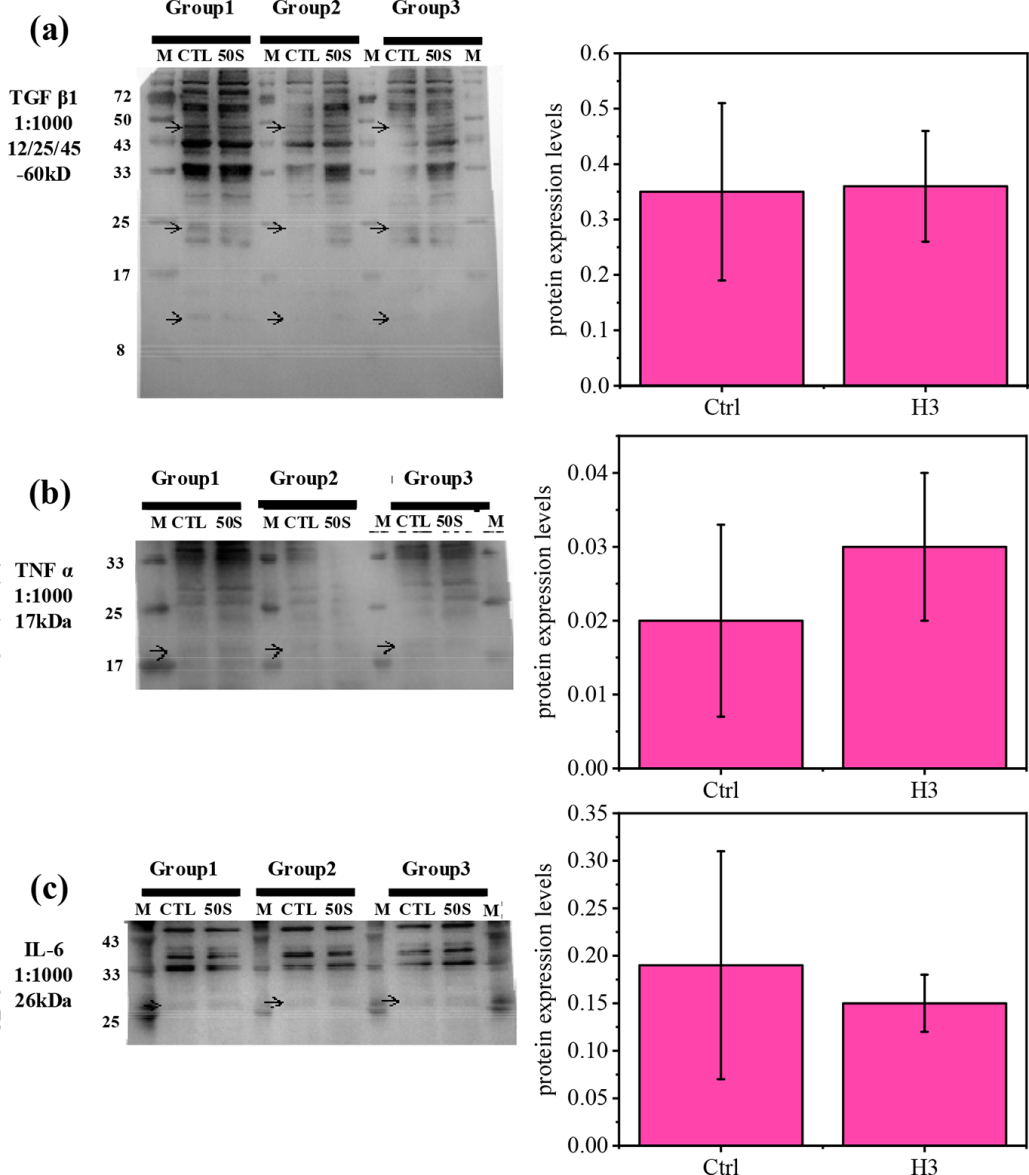
(a) TGF-β, (b) TNF-α, and (c) IL-6 protein expression levels in the control and H3 groups. Arrows indicate the specific locations of the proteins.

## 4 Conclusions

This study systematically explored the potential of hydro-amphiphilic ternary copolymers, comprising hydrophilic hydroxyethyl methacrylate (HEMA), hydrophobic styrene, and poly­(ethylene glycol) phenyl ether acrylate (PEGPEA), as promising materials for intraocular lenses (IOLs). By carefully adjusting monomer ratios, these copolymers were tailored to meet essential performance benchmarks, including mechanical robustness, foldability, optical clarity, biocompatibility, and durability. Hydrophobic styrene contributed to enhanced stiffness and a higher refractive index but reduced break strain. This drawback was mitigated by the inclusion of hydrophilic HEMA, which also increased water uptake and prevented glistening, while PEGPEA balanced the opposing properties to create an optimized material profile. As a proof of concept, the H3 formulation, featuring a well-balanced hydrophilic-to-hydrophobic composition, demonstrated optimal mechanical properties, including a low Young’s modulus, high break strain, and superior foldability, making it ideal for minimally invasive implantation. This material also effectively resisted glistening and preserved optical transparency during aging tests, with no evidence of microvacuole formation. Biocompatibility evaluations confirmed low cytotoxicity, and inflammation levels were comparable to those of a commercially available IOL. These results highlight the promise of hydro-amphiphilic copolymers as a next-generation platform for IOL materials, offering a foldable, glistening-free, and biocompatible solution that enhances both surgical outcomes and long-term patient satisfaction.

## Supplementary Material



## Notes

FTIR-ATR spectra of IOL materials (H3, P3, S3), optical microscopy images demonstrating material transparency, primer sequences used for qPCR analysis of inflammatory gene expression in HLEB3 cells (PDF)
