# Supplementary material for: Strategic Design of Biocompatible, Glistening-Free, and Foldable Artificial Intraocular Lenses Based on Hydro-Amphiphilic Ternary Copolymers
Source: Biomacromolecules. 2025 Jun 17;26(7):4612–25. doi: 10.1021/acs.biomac.5c00588 (PMC12264951; doi:10.1021/acs.biomac.5c00588)
Supplement: Supplementary file 1 [file bm5c00588_si_001.pdf]

SUPPORTING INFORMATION

Strategic Design of Biocompatible, Glistening-Free and Foldable Artificial Intraocular Lenses Based on Hydro-Amphiphilic Ternary Copolymers

Cheng-Ti Hu,[1] Zhi-Xuan Liang,[1] Jhen-Yu Luo,[2] Po-Hsun Chiu,[1] Annabelle I. Day,[5] Chih-Chen Hsieh,[1] Po-Jen Shih,[3] Jia-Han Li,[4] Bo-I Kuo,[6,*] I-Jong Wang,[6,7] Jia-Yush Yen,[8] and Chi-An Dai,[1,2,*]

[1]Department of Chemical Engineering, [2]Institute of Polymer Science and Engineering, [3]Department of Biomedical Engineering, and [4]Department of Engineering Science and Ocean Engineering, National Taiwan University, Taipei, 10617, Taiwan

[5]Institute of Neuroscience, National Yang-Ming Chiao-Tung University, Taipei, 112304, Taiwan

[6]Department of Ophthalmology, National Taiwan University Hospital, Taipei, 100225, Taiwan

[7]College of Medicine, National Taiwan University, Taipei, 100233, Taiwan

[8]Department of Mechanical Engineering, National Taiwan University of Science and Technology, Taipei, 106335 Taiwan

*Corresponding authors. E-mail: bikuo0514@gmail.com (B.-I. Kuo) polymer@ntu.edu.tw (C.-A. Dai)

ADDITIONAL TABLES

Table S1. Primer sequences used for qPCR analysis of inflammatory gene expression in HLEB3 cells cultured on IOL samples

Gene Primer	Sequence (5'-3')
ACTB-F	ATGTGCAAGGCCGGCTTC
ACTB-R	GAATCCTTCTGACCCATGCC
TGFB1-F	CAAGGGCTACCATGCCAACT
TGFB1-R	GGTTATGCTGGTTGTACAGGGC
TNF-F	GAGTGACAAGCCTGTAGCCCA
TNF-R	TCTCTCAGCTCCACGCCATT
IL6-F	GAGGCACTGGCAGAAAACAAC
IL6-R	CAAACTCCAAAAGACCAGTGATGA

ADDITIONAL FIGURES

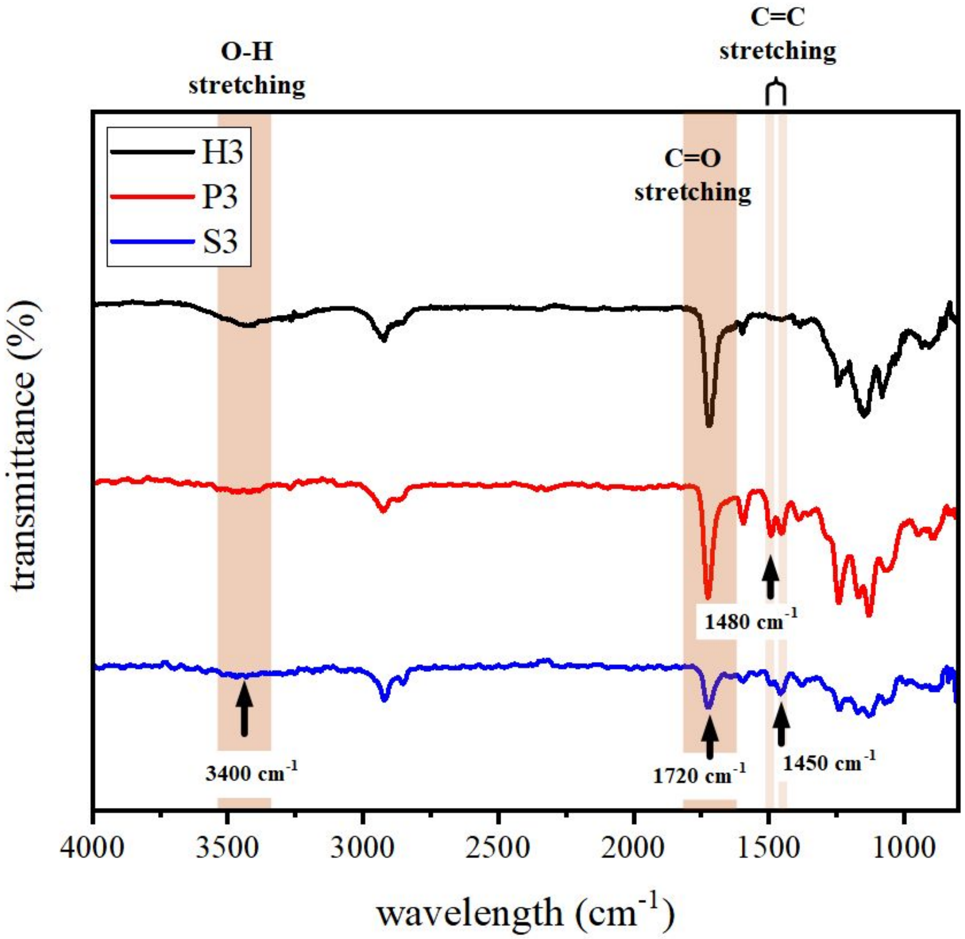

Figure S1. FTIR-ATR spectra of representative IOL samples (H3, P3, and S3), highlighting characteristic absorption bands that reflect compositional differences among the monomers. Key peaks corresponding to distinctive functional groups in each monomer are shaded in light orange for easier comparison.

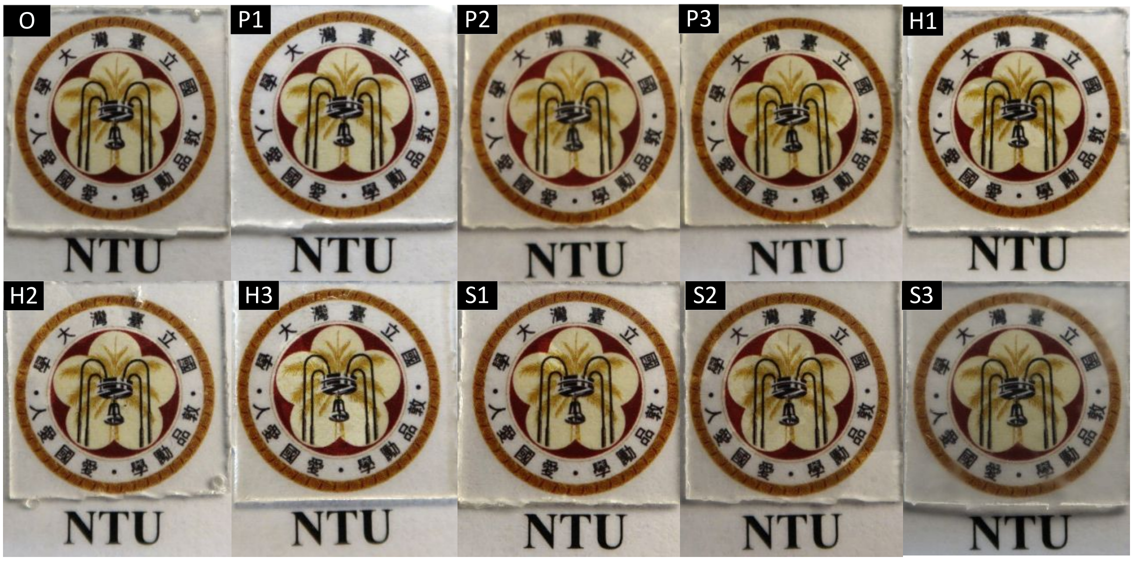

Figure S2. Optical microscope images demonstrating the optical clarity of the tested IOL materials. The NTU emblem shown in the background is used with permission from National Taiwan University.
